# Perceived psychosocial impairment associated with eating disorder features: responses to a mental health literacy intervention

**DOI:** 10.1186/s40337-015-0084-9

**Published:** 2015-12-02

**Authors:** Caroline Bentley, Kassandra Gratwick-Sarll, Jonathan Mond

**Affiliations:** Research School of Psychology, Australian National University, Canberra, ACT 0200 Australia; Department of Psychology, Macquarie University, Sydney, Australia

**Keywords:** Eating disorder features, Psychosocial functioning, Mental health literacy

## Abstract

**Background:**

Whether and to what extent young adults are aware of the adverse impact of eating disorder features (EDF) on psychosocial functioning is unclear, although such awareness may affect the experience and behavior of sufferers. The aim of the current study was to examine young adults’ perceptions of psychosocial impairment associated with EDF, and the potential effect on these perceptions of an eating disorders “mental health literacy” (ED-MHL) intervention.

**Methods:**

Undergraduate students (male: *n* = 35; female: *n* = 141) completed self-report questionnaires prior to, immediately following, and 3 months after completion of a 3-h ED-MHL intervention. Perceived psychosocial impairment associated with EDF–binge eating, purging, extreme dietary restriction, overvaluation of weight/shape, and excessive exercise–was assessed at each time point.

**Results:**

At all 3 time points, EDF were considered to have a ‘slightly negative’ to ‘very negative’ impact on psychosocial functioning. Prior to the intervention, binge eating, purging and extreme dietary restriction were generally considered to have a greater negative impact than excessive exercise and overvaluation of weight/shape. Three months after the ED-MHL intervention, participants reported greater perceived impairment associated with excessive exercise and overvaluation; while perceptions of psychosocial impairment associated with binge eating, purging and dietary restriction remained largely unchanged. Females perceived greater impairment associated with EDF than males did immediately after the intervention, but not at the 3-month follow-up.

**Conclusions:**

The adverse effects on psychosocial functioning of binge eating, purging and extreme dietary restriction appear to be readily recognized by young people. Awareness of the adverse effects of excessive exercise and overvaluation may be poorer, but amenable to improvement by means of a relatively simple intervention. These features may warrant particular attention in health promotion programs.

## Background

In recent years, evidence has accumulated concerning the negative impact of eating disorder features, namely, binge eating, extreme weight-control behaviors, and key cognitive features, such as the overvaluation of body weight/shape, on individuals’ quality of life [1–3]. Increasing focus on eating disorder features (EDF) is appropriate, given broad acceptance of the transdiagnostic model of eating disorder psychopathology [4] and increasing awareness of the need for a dimensional, symptom-based approach to diagnostic criteria for mental disorders [5, 6]. Assessing EDF rather than eating disorders can provide more stable construct definitions over time and accommodate individuals who might otherwise be excluded by virtue of overly restrictive diagnostic criteria [5, 6]. For these reasons, it is increasingly recognized that reducing the adverse impact of EDF on individuals’ quality of life should be a key target of preventive interventions [6–8].

One way to reduce the adverse impact of EDF on individuals’ quality of life is to improve public awareness and understanding of the nature of these features and of the distress and disability that they engender [9]. Improving these and other aspects of eating disorders “mental health literacy” (ED-MHL) would have, potentially, at least two benefits. First, improved community awareness and understanding of the nature and adverse impact of EDF may be conducive to improved uptake of mental health care, where this is needed, among sufferers [10–12]. Improved uptake of mental health care would be expected to follow from improvements in the ED-MHL not only of individuals who have or may develop symptoms, but also of these individuals’ family and friends and members of their social networks more generally [9, 13, 14]. Evidence suggests that appropriate interventions may be effective in improving awareness and understanding of the nature and treatment of mental health problems as well as confidence in assisting–and willingness to assist–sufferers to seek appropriate help, including interventions designed to improve ED-MHL [13, 15]. Second, improved awareness of the nature and adverse impact of EDF may be conducive to lower levels of stigma towards, and, in turn, among sufferers. Lower levels of internalized stigma would be expected to have a direct benefit in terms of improved quality of life, while also being conducive to improved uptake of mental health care where this is needed [16].

Available evidence suggests that the public is generally sympathetic toward individuals with eating disorders–anorexia nervosa and bulimia nervosa at least–in that people generally recognize the seriousness and distressing nature of these conditions [17]. However, it is also apparent that a substantial minority (around one third) of people, young adults in particular, hold attitudes that may trivialize and otherwise stigmatize eating disorders, such as the beliefs that having an eating disorder ‘might not be too bad’ [17] and that individuals with an eating disorder ‘only have themselves to blame’ and should ‘pull themselves together’ [18]. Further, attitudes of this kind may be more common in young men than in young women [19, 20].

There is some evidence that individuals’ beliefs about the adverse impact of eating disorders vary as a function of the features comprising the disorder [19–21]. Thus, the perception that eating disorders are mild or even trivial may be more commonly ascribed to individuals with binge eating disorder than individuals with anorexia nervosa or bulimia nervosa [19, 21]. That is, in the absence of extreme weight-control behaviors and/or low body weight, the adverse impact of binge eating may be underestimated. Perceived impairment associated with “non-purging” extreme weight-control behaviors, namely, extreme dietary restriction and excessive exercise, may also tend to be underestimated, given that these behaviors are more “normative” and socially sanctioned, whereas the distress caused by purging behaviors may be more likely to be recognized [22, 23]. The negative impact of weight/shape overvaluation may also be underestimated, given the overlap of this construct with body dissatisfaction [24], despite its status as a core feature of eating disorder psychopathology [4] and despite evidence suggesting it may be a better predictor of mental health impairment than other EDF [7, 8].

To our knowledge, no study has examined individuals’ perceptions of impairment associated with different EDF. Research of this kind may be helpful in indicating which EDF may be more or less susceptible to trivialization. Ideally, research of this kind would be conducted in young adults, given that eating disorders–and EDF–typically have their onset in adolescence or early adulthood [5]. Further, it would be helpful not only to document young adults’ perceptions of the impairment associated with EDF, but also to determine whether a brief intervention might be effective in improving this aspect of ED–MHL, should it be found to be problematic. The goal of the current study was, therefore, to examine young adults’ perceptions of psychosocial impairment associated with EDF prior to and following an ED-MHL intervention. A secondary aim of the study was to examine sex differences in perceptions of impairment over time. In view of the paucity of existing evidence, our only a priori hypotheses were that purging behaviors would be seen to be associated with greater impairment in psychosocial functioning than non-purging behaviors and that young men would perceive at least some EDF to be associated with lower levels of impairment than young women at one or more time points.

## Method

### Study design and recruitment of participants

Participants were 141 female and 35 male (*N* = 176) third-year undergraduate psychology students from the Australian National University who opted in to the voluntary research component of the ‘Should I Say Something?’ workshop, which was offered through a laboratory class. Of the students enrolled in the laboratory classes, 94.6 % volunteered to participate in the study at baseline. The dropout rate across time was 8.0 %. ‘Should I Say Something?’ is a 3-h interactive workshop developed by Hart et al. [13], with promising preliminary findings regarding its effectiveness in promoting ED-MHL [15]. The workshop is designed to improve participants’ understanding of the nature and treatment of eating disorders and to provide them with the skills to assist someone close to them (or themselves) to seek early and appropriate treatment for an eating disorder or other mental health issue. The workshop was conducted by the first and second author and included a slide presentation, a vignette on the lived experience of a young woman with anorexia nervosa, and individual and group activities throughout (cf. [13, 15]). No remuneration or other incentives to participation were provided. Participants completed questionnaires immediately before and after participating in the workshop (Time 1 and Time 2, respectively) and at a 3-month follow-up (Time 3). Questionnaires assessed participants’ perceptions of psychosocial impairment associated with different EDF; as well as ascertaining participants’ socio-demographic information and experience with eating disorders (Table 1). The questionnaires also included other measures not relevant to the present study which are detailed elsewhere [15]. The vast majority of participants (98 %) were aged 18 to 26 years at baseline. Participants reported that 31.8 % had studied eating disorders at university and 42.0 % believed they had known someone personally with an eating disorder. The study design and methods were approved by the Australian National University Human Research Ethics Committee (protocol no. 2012/523).

**Table 1:** Demographic characteristics of study participants (Time 1)

	Women	Men	Total Sample
	(*n* = 141)	(*n* = 35)	(*N* = 176)
	%	%	%
Sex	80.1	19.9	100
Born in Australia	61.7	65.7	62.5
English as first language	75.2	82.9	76.7
Studied eating disorders at university	31.9	31.4	31.8
Knew/knows someone with an eating disorder	44.0	34.3	42.0
	Mean (SD)	Mean (SD)	Mean (SD)
Age (years)	20.00 (2.90)	21.09 (4.79)	20.22 (3.38)

### Study measures

Perceived psychosocial impairment associated with different EDF was assessed by asking participants: “How do you think [a specific EDF] would impact on someone's [psychosocial functioning]?” Psychosocial functioning was defined by two items which were subsequently combined: “emotional wellbeing” and “social relationships (with friends, family & partners)”. Participants responded to each item on a five-point, Likert-type scale (1 = ‘very positive impact’; 2 = ’slightly positive impact’; 3 = ’impact neither positive nor negative’; 4 = ‘slightly negative impact’; 5 = ‘very negative impact’). Cronbach’s alphas in the present study for psychosocial functioning (Time 1) were 0.76 for female participants and 0.75 for male participants.

Specific EDF were defined as follows. Purging was defined as “Inducing vomiting, weekly or more, in order to influence one's body weight and/or shape” and/or " Taking laxatives, weekly or more, in order to influence one’s body weight and/or shape”. Extreme dietary restriction was defined as “Going for long periods of time (8 h + during the day) without eating anything, on most days, in order to influence one’s body weight and/or shape” [25, 26]. Binge eating was defined as “Eating what other people would think was a very large amount of food given the situation and feeling a loss of control over eating at the time, weekly or more” [5]. Excessive exercise was defined as “Exercising really hard in a driven or compulsive way, on most days, in order to influence one’s body weight and/or shape” [25, 26]. Overvaluation of weight/shape was defined as “Thinking one’s body weight and/or shape is very important to their self-esteem”.

### Statistical analysis

Data were stratified by sex for the purpose of descriptive statistics (mean ratings of perceived impairment associated with each EDF at each time point) and in all analyses. Tests of significant differences between ratings of different EDF at each time point, and across different time points were conducted using Friedman tests and post-hoc Bonferroni-corrected Wilcoxon Signed Rank tests. Mann Whitney U tests were employed to test for sex differences in ratings for each EDF at each time point. A significance level of .05 was adopted for all tests where a Bonferroni correction is not specified. All analysis was conducted using SPSS version 22.0.

## Results

**Fig. 1:**
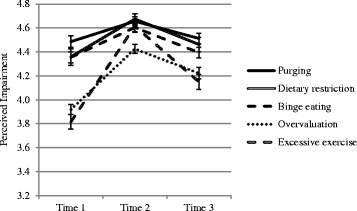
Mean ratings of perceived impairment associated with eating disorder features across time points (females)

**Fig. 2:**
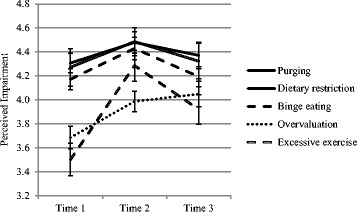
Mean ratings of perceived impairment associated with eating disorder features across time points (males)

Mean ratings of perceived psychosocial impairment associated with different EDF across the three time points are presented in Fig. 1 (females) and Fig. 2 (males). It can be seen that all mean ratings-across time points and for both sexes-reflect perceptions of EDF as having a ‘slightly negative’ to ‘very negative’ impact on psychosocial functioning (i.e., mean range is 3.5 to 4.7).

### Perceived impairment associated with EDF

Friedman tests of differences in mean ratings of perceived impairment associated with different EDF (for both sexes, at three time points) were all significant (all *p* < .01). Post-hoc Bonferroni-corrected Wilcoxon Signed Rank tests identified the source of these significant differences, which are reported in text for the sake of brevity. At Time 1, females’ mean ratings of impairment associated with purging, dietary restriction and binge eating were significantly higher than their mean ratings associated with overvaluation and excessive exercise (all *p* < .001). For males at Time 1, mean ratings of impairment associated with purging and dietary restriction were significantly higher than mean ratings associated with overvaluation and excessive exercise; mean ratings associated with binge eating were significantly higher than mean ratings associated with excessive exercise (all *p* < .001).

At Time 2, females’ mean ratings of impairment associated with purging, dietary restriction, binge eating and excessive exercise were significantly higher than mean ratings associated with overvaluation (all *p* < .005). For males at Time 2, mean ratings of impairment associated with purging, dietary restriction and binge eating were significantly higher than mean ratings associated with overvaluation (all *p* < .005).

At Time 3, females’ mean ratings of impairment associated with purging, dietary restriction and binge eating were significantly higher than mean ratings associated with excessive exercise; mean ratings associated with purging and dietary restriction were significantly higher than mean ratings associated with overvaluation (all *p* < .005). For males at Time 3, mean ratings of impairment associated with purging and dietary restriction were significantly higher than mean ratings associated with excessive exercise (all *p* < .005).

### Change over time in perceived impairment associated with EDF

Results of Friedman tests of differences across time for mean ratings of perceived impairment associated with specific EDF, by sex, are presented in Table 2. Results of post-hoc Bonferroni-corrected Wilcoxon Signed Rank tests are also presented in Table 2, to identify the source of significant differences (where applicable). It can be seen that females’ mean ratings of impairment associated with all five EDF significantly increase immediately post intervention, then significantly decrease by 3-month follow-up. This resulted in no significant difference between baseline and follow-up for female ratings of impairment associated with purging, dietary restriction and binge eating; while female ratings of impairment associated with overvaluation and excessive exercise remained significantly higher at follow-up than baseline.

**Table 2:** Results of Friedman tests and post-hoc Wilcoxon signed rank tests (where applicable) for perceived impairment associated with eating disorder features (across time points, split by sex)

		Friedman	Time 1 *vs*. Time 2	Time 2 *vs*. Time 3	Time 1 *vs*. Time 3
		χ^2^	z	z	z
Females	Purging	15.29**	−3.92**	−3.19**	−0.71
	Dietary restriction	44.57**	−6.17**	−4.70**	−1.35
	Binge eating	28.66**	−5.04**	−4.15**	−0.68
	Overvaluation	54.45**	−7.10**	−3.51**	−4.06**
	Excessive exercise	111.55**	−8.86**	−6.31**	−4.51**
Males	Purging	3.66	-	-	-
	Dietary restriction	4.88	-	-	-
	Binge eating	5.49	-	-	-
	Overvaluation	7.34*	−2.13*	−0.22	−2.13*
	Excessive exercise	19.60**	−4.55**	−2.01*	−2.51*

* < .05; ** < .001

For males, there were no significant changes across time points for ratings of impairment associated with purging, dietary restriction and binge eating. For perceived impairment associated with excessive exercise, males significantly increased their ratings between baseline and post intervention (*p* < .001) and sustained this change (relative to baseline) at 3-month follow-up (*p* = .012). For perceived impairment associated with overvaluation, males appeared to increase their ratings between baseline and post intervention and sustain this change at follow-up, however these results were not significant (both *p* = .033; Bonferroni-corrected α = .017).

### Sex differences in perceived impairment associated with EDF

Sex differences in ratings of perceived psychosocial impairment associated with different EDF at each of the three time points are presented in Table 3. As can be seen, at Times 1 and 3 there were no significant sex differences regarding participants’ ratings of the perceived psychosocial impairment associated with EDF. At Time 2, female participants rated psychosocial impairment associated with EDF as significantly greater than male participants did for purging (*p* < .05), dietary restriction (*p* < .05), overvaluation (*p* < .01) and excessive exercise (*p* < .01).

**Table 3:** Results of Mann Whitney U tests of sex differences in mean scores for perceived impairment associated with eating disorder features across time points

	Time 1 *N* = 141 (f) + 35 (m)		Time 2 *N* = 140 (f) + 35 (m)		Time 3 *N* = 131 (f) + 31 (m)	
	z	r^i^	z	r	z	r
Purging	−1.87	0.14	−1.99*	0.15	−1.50	0.11
Dietary restriction	−1.25	0.09	−2.01*	0.15	−1.24	0.09
Binge eating	−1.87	0.14	−1.79	0.13	−1.95	0.15
Overvaluation	−1.58	0.12	−3.42**	0.26	−1.58	0.12
Excessive exercise	−1.81	0.14	−2.79**	0.21	−1.59	0.12

* < .05; ** < .01

^i^Small effect size = .1; medium effect size = .3; large effect size = .5

## Discussion

### Summary of main findings

This study examined individuals’ perceptions of psychosocial impairment associated with EDF and the potential effects of an ED-MHL intervention on these perceptions. EDF included binge eating, purging, extreme dietary restriction, excessive exercise and overvaluation of weight/shape. Prior to the intervention, participants reported, on average, perceptions of each EDF as having a ‘slightly negative’ to ‘very negative’ impact on psychosocial functioning. Generally, binge eating, purging and dietary restriction were considered to have a greater adverse impact on psychosocial functioning than excessive exercise and overvaluation. None of these findings differed by sex. Immediately following the intervention, females’ ratings of perceived impairment associated with EDF were higher than males’ ratings for all EDF except binge eating. Females’ ratings of perceived impairment associated with EDF increased for all five EDF, whereas males’ ratings increased only for excessive exercise. At the 3-month follow-up, perceptions of impairment associated with binge eating, purging and dietary restriction did not differ from those observed at baseline, whereas perceptions of impairment associated with excessive exercise were greater than at baseline. In females, but not males, perceptions of impairment associated with overvaluation were greater at the 3-month follow-up than at baseline. No sex differences were observed at the 3-month follow-up.

### Study implications

Several of these findings are encouraging. First, it is encouraging that participants generally perceived the EDF considered to have a negative impact on psychosocial functioning. Second, it is encouraging that the degree of impairment attributed to EDF was similar among male and female participants. This finding contrasts with those of previous studies suggesting that young men are more prone to trivialize eating disorders than young women [19, 20]. Third, it is encouraging that participants appeared to be aware that dietary restriction may be associated with impairment in psychosocial functioning comparable to that of binge eating and purging. Concerns have been expressed that impairment associated with extreme dietary restriction (e.g., [7, 27]) may be minimized due to the fact that it may be seen to be “normative” or even desirable, particularly in the context of concerns surrounding the “obesity epidemic” [28]. Fourth, it is encouraging that participants’ perceptions of impairment associated with excessive exercise and overvaluation increased following the ED-MHL intervention, and that pre-post differences in this regard were still apparent at the 3-month follow-up. This finding adds to a small but growing literature on the utility of ED-MHL interventions [13, 15].

Certain other findings were, however, less encouraging. First, the benefits of the intervention in terms of increasing awareness of the negative impact were largely confined to female participants. The content of the intervention (e.g., vignette depicting only a female sufferer), the sex of the presenters (both female) and female participants being more prone to social desirability effects [29], may all have been factors in this regard. Second, these differential benefits appeared to be short-lived, since sex differences were no longer apparent at the 3-month follow-up, although as noted above some changes were maintained at follow-up by both males and female participants. The tendency for post-intervention changes in knowledge and beliefs concerning eating-disordered behavior to diminish over time, which was observed in early studies of eating disorder prevention trials [30], highlights the importance of including follow-up assessments and, perhaps, the need for booster sessions, in future trials of this kind. Third, perceptions of impairment associated with overvaluation and excessive exercise were lower than for other EDF at baseline and, to a lesser degree, at follow-up. Although this finding may not be surprising, it is concerning given that overvaluation is a core component of eating disorder psychopathology [4] and known to be strongly associated with impairment in mental health and quality of life in both males and females [3, 7, 8]. Findings relating to perceived impairment associated with excessive exercise are more difficult to interpret, given the lack of any agreed-upon operational definition of this term [28], but there is good evidence that certain exercise behaviors, such as exercising primarily as means of controlling weight or shape, are strongly associated with eating disorder psychopathology and, in turn, impairment in quality of life, in young women [31]. Further, there is growing concern about the adverse physical and mental health consequences of excessive exercise–that aimed at developing muscle mass in particular–in young men [6]. Hence, efforts may be needed to improve community ED-MHL relating to the adverse effects of overvaluation and excessive exercise in particular.

### Study strengths and limitations

A limitation of the current study is the relatively small number of male participants (*n* = 35) and, in turn, relatively lower statistical power to detect effects involving males and differences between males and females. The use of psychology students as participants might also be considered a limitation of the current study, given the greater potential for ceiling effects regarding ED-MHL in this population and other characteristics of student samples that may limit the generalizability of the findings to the broader population. On the other hand, interventions of this kind may be particularly important among psychology students and in student populations more generally, given that EDF and other mental health problems may be over-represented in these populations [32–34]. Strengths of the current study include the recruitment of male and female participants, assessment of perceptions of impairment associated with a broad range of EDF, assessment immediately following and 3 months following the intervention, and the low rates of dropout. This is, to our knowledge, the first study to examine individuals’ perceptions of psychosocial impairment associated with different EDF and potential changes in these perceptions in response to an ED-MHL intervention.

## Conclusion

Findings from the current study suggest that ED-MHL concerning awareness of the adverse effects of EDF on quality of life among male and female psychology students may generally be acceptable. Adverse effects of extreme dietary restriction, binge eating and purging at least appear to be readily recognized in this population. Awareness of the adverse effects of excessive exercise and overvaluation was initially poorer, but improved somewhat following an ED-MHL intervention. These features may warrant particular attention in health promotion programs. Future trials of ED-MHL interventions may benefit from the inclusion of vignettes depicting male sufferers and/or male facilitators.
